# Reply to letter in response to Rethinking the diagnosis of double‐seronegative myasthenia gravis

**DOI:** 10.1111/ene.16152

**Published:** 2023-11-22

**Authors:** Rodrigo Martinez‐Harms, Carolina Barnett, Monica Alcantara, Vera Bril

**Affiliations:** ^1^ Ellen & Martin Prosserman Centre for Neuromuscular Diseases Toronto General Hospital Toronto Ontario Canada; ^2^ University Health Network, University of Toronto Toronto Ontario Canada

Dear Editor,

We wish to thank Drs. Seok, Kim, Eun, and Lee for their valuable comments about our recently published original article “Clinical Characteristics and Treatment Outcomes in Patients with Double‐Seronegative Myasthenia Gravis” [1]. We are very glad to see that our paper is stimulating further discussion on the topic of patients without detectable antibodies to nicotinic acetylcholine receptor (AChR) and muscle‐specific kinase. The authors correctly point out that we found a significant clinical improvement in double‐seronegative myasthenia gravis (dSNMG) patients in the last clinical evaluation, based on the Myasthenia Gravis Impairment Index and Single Simple Question [2, 3] scores strongly suggesting an immune‐based mechanism and the benefit of immunotherapy in this group of patients.

dSNMG represents a heterogeneous group, and the diagnosis is challenging and commonly based on the clinical presentation and progression. It is fundamental to exclude differential diagnoses such as congenital myasthenia gravis, muscular dystrophies, or other disorders of neuromuscular transmission. The authors think that the electrophysiological findings based on single fiber electromyography and repetitive nerve stimulation are essential in the diagnosis of myasthenia gravis (MG) [4]. There are additional complementary diagnosis tests for MG, for example, the edrophonium and neostigmine tests, which can be reliable, but are rarely used in clinical practice. Therefore, we did not use these tests as an inclusion criterion in our study. We agree fully with Seok et al. that patients who have refractory seronegative MG should be extensively reviewed for the possibility of alternate diagnoses, although we understand that refractoriness alone does not exclude a diagnosis, as seropositive MG patients can be refractory to treatment.

There is a need for further characterization of serological markers in seronegative MG patients. Novel antibodies have been associated with MG and could be present in this group of patients. Especially relevant is the lipoprotein receptor‐related protein 4 (LRP4), which may be an MG marker. One weakness of our study is the absence of LRP4 antibody serology characterization, which might have reduced the numbers considered to be dSNMG. Additional antibodies against other extracellular or intracellular targets have been found in some MG patients, such as agrin, collagen Q, Kv 1.4 potassium channels, titin, the ryanodine receptor, and cortactin, and these may play a role in MG [5]. Nevertheless, whether these antibodies produce disease pathology or are just an epiphenomenon needs to be further studied. Some of these antibodies have been found in healthy controls or associated with other immune conditions like cortactin antibodies, present in 20% of those with poliomyelitis [4]. It may be that dSNMG patients have one of these potential new markers, and this association hypothetically might be associated with a variation in the classic MG treatment response and have a poor response compared to classic AChR‐positive MG.

In conclusion, dSNMG patients have significant clinical improvement after treatment, supporting an immune‐mediated pathophysiology and encouraging efforts to improve the response to treatment. The development of new biomarkers is promising and may improve personalized clinical characterization and individualized therapeutic approaches.

## AUTHOR CONTRIBUTIONS

**Rodrigo Martinez‐Harms:** Conceptualization; writing – original draft; validation; writing – review and editing; supervision; project administration; investigation. **Carolina Barnett:** Conceptualization; writing – review and editing; supervision. **Monica Alcantara:** Conceptualization; writing – review and editing; supervision. **Vera Bril:** Conceptualization; writing – review and editing; supervision; project administration; writing – original draft.

## CONFLICT OF INTEREST STATEMENT

None of the authors has any conflict of interest to disclose.

## Data Availability

The data that support the findings of this study are available on request from the corresponding author. The data are not publicly available due to privacy or ethical restrictions.
